# Orbital Ectopic Lymphoid Follicles with Germinal Centers in Aquaporin-4-IgG-Positive Neuromyelitis Optica Spectrum Disorders

**DOI:** 10.3389/fimmu.2017.01947

**Published:** 2018-01-16

**Authors:** Koon Ho Chan, Raymand Lee, Kui Kai Lau, Florence Loong

**Affiliations:** ^1^Department of Medicine, LKS Faculty of Medicine, The University of Hong Kong, Hong Kong, Hong Kong; ^2^Neuroimmunology and Neuroinflammation Research Laboratory, LKS Faculty of Medicine, The University of Hong Kong, Hong Kong, Hong Kong; ^3^Research Center of Heart, Brain, Hormone and Healthy Aging, LKS Faculty of Medicine, The University of Hong Kong, Hong Kong, Hong Kong; ^4^Department of Diagnostic Radiology, LKS Faculty of Medicine, The University of Hong Kong, Hong Kong, Hong Kong; ^5^Department of Pathology, LKS Faculty of Medicine, The University of Hong Kong, Hong Kong, Hong Kong

**Keywords:** neuromyelitis optica spectrum disorder, aquaporin 4, autoimmunity, ectopic lymphoid structures, germinal center

## Abstract

Neuromyelitis optica spectrum disorders (NMOSDs) are important autoimmune central nervous system (CNS) astrocytopathy causing acute myelitis, optic neuritis (ON), and encephalitis associated with significant morbidities and mortality. It is important to diagnose NMOSDs early as they are treatable. The majority of NMOSDs patients are seropositive for aquaporin-4 IgG (AQP4-IgG) autoantibodies, which target CNS aquaporin-4 (AQP4) expressed abundantly in astrocytic foot processes. We report the novel observation of orbital masses containing ectopic lymphoid follicles with germinal centres (GC) in two patients with AQP4-IgG-positive NMOSD. Both patients had severe extensive myelitis with symptomatic or asymptomatic ON, with the ectopic lymphoid structures detected on initial presentation. Histolopathological studies confirmed that the orbital masses contained reactive lymphoid follicles with GC containing B cells and plasma cells. Our observations support that AQP4-IgG positive NMOSDs patients have underlying AQP4 autoimmunity and suggest that ON (symptomatic or asymptomatic) may trigger formation of orbital ectopic GC contributing to development of high-affinity AQP4-specific memory B cells and plasma cells, which produce highly pathogenic AQP4-IgG.

## Background

Neuromyelitis optica spectrum disorders (NMOSD) are central nervous system (CNS) inflammatory disorders (1–3). Relapsing acute neuroinflammation is the clinical course in the majority of NMOSD patients characterized by recurrent attacks of unilateral or bilateral optic neuritis (ON) and acute myelitis, typically longitudinally extensive transverse myelitis (LETM) (1, 2). The diagnosis of NMOSD are greatly facilitated by detection of IgG autoantibodies targeting aquaporin-4 (AQP4) (1, 4–6). Aquaporin-4 IgG autoantibodies (AQP4 IgG) are detected in the serum of about 75% of patients and are specific for NMOSD (1, 4). AQP4 is a water channel highly expressed in astrocytic endfoot processes and plays important roles in CNS water homeostasis (7).

Neuromyelitis optica spectrum disorders patients seropositive for AQP4-IgG have underlying autoimmunity targeting AQP4 (1, 2). The pathogenetic mechanisms triggering AQP4 autoimmunity is uncertain, but B cells certainly play important roles (8). CD19^+^CD27^high^CD38^high^CD130^−^ plasmablasts are increased in peripheral blood of NMO patients, which secrete AQP4-IgG under stimulation by IL-6 (9), and anti-CD20 monoclonal antibody (rituximab) is effective in some NMOSD patients (10). Germinal centers (GC) in secondary lymphoid organs are important sites for production of high-affinity specific antibodies in adaptive immunity and high-affinity pathogenic autoantibodies in autoimmune diseases. Interestingly, ectopic lymphoid follicles (ELF) containing GC are observed in target tissues in autoimmune diseases such as lymphofollicular hyperplasia in thymuses of early onset myasthenia gravis (11).

## Case Presentation, Laboratory Investigations, and Diagnostic Tests

### Patient 1

A 67-year-old Chinese woman had steroid-refractory immune thrombocytopenic purpura treated with splenectomy, hypertension, diabetes, bilateral glaucoma on regular eyedrops for 7 years and benign thyroid tumor resected 8 years ago. She developed radicular pain at T6–8 dermatomes followed by bilateral lower limb paresthesia and numbness ascending to T2 dermatome, paraplegia, and urinary retention over 2 weeks. She had upper respiratory tract infection 2 weeks before onset of lower limb symptoms. Physical examination confirmed paraplegia, upgoing plantars, and sensory level at T6. MRI spine revealed T2W hyperintensity consistent with LETM affecting C6-T6 with mild contrast enhancement. CSF analysis revealed mild pleocytosis of 15 × 10^6^/L (48% neutrophil, 18% lymphocyte, 29% mononuclear cells, and 5% eosinophil), normal protein, and glucose levels without oligoclonal bands or malignant cells. She was treated with intravenous methylprednisolone (IVMP) 1 g daily for 5 days and gradually improved to walking with frame 4 weeks later. Right visual-evoked potential (VEP) latency was prolonged to 128 ms and left VEP was normal. Her serum IgG level was elevated to 2,590 mg/dL with normal IgA and IgM levels, but no monoclonal band was detected. She was seropositive for antinuclear antibody (ANA) at 1/40 with minimally elevated anti-ds DNA at 37 IU/mL, but seronegative for NMO-IgG (tissue-based immunofluorescence).

**Figure 1:**
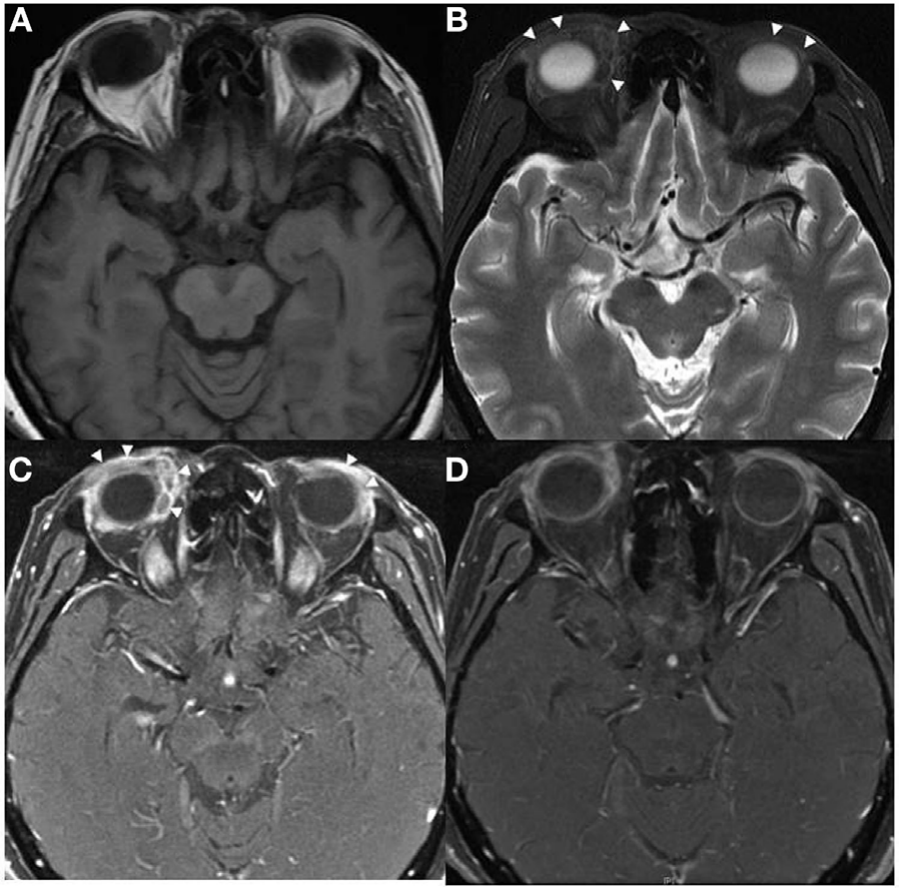
Axial MRI of patient 1 showed predominately extraconal infiltrating soft tissue lesions in both orbits (arrowheads) with significant preseptal component, they were hypointense on T1W **(A)** and T2W **(B)** images with heterogeneous contrast enhancement **(C,D)**.

She reported right eye puffiness for 2 years, which was reduced in severity after pulse steroid therapy for myelitis. Ophthalmologist’s assessment revealed a right conjunctival mass. MRI brain and orbits performed 7 weeks later revealed no cerebral abnormalities, but a T1 and T2 hypointense lesion surrounding the superior aspect of the right eyeball with heterogeneous, predominantly peripheral contrast enhancement, and the left eyeball was similarly affected to a lesser extent while the optic nerves and extraocular muscle were unremarkable (Figure 1). Lymphoma was suspected but she had no palpable lymph node or organomegaly. Right conjunctival excisional biopsy performed 3 months after myelitis onset revealed a circumscribed lymphoid lesion consisting of hyperplastic secondary follicles with small lymphocytes and plasmacytosis associated with prominent interfollicular vasculature. The plasma cells were mature looking, and there was no epithelial component. Immunohistochemstry confirmed that the GC were BCL2 negative supporting they were reactive in nature, and the interfollicular regions contained moderate number of T lymphocytes; the plasma cells were polytypic for kappa and lambda light chain with a normal ratio. Cytokeratin MNF116 staining confirmed the absence of epithelial element. These were suggestive of reactive lymphoid hyperplasia.

Three months later, she developed right homonymous hemianopia followed by bilateral lower limb weakness and urinary retention over 6 days. MRI spine showed recurrent myelitis lesion over C2-T6. MRI brain showed inflammatory demyelinating lesions involving the deep and periventricular white matter around the left posterior temporal and occipital horn, and part of the splenium of corpus callosum, mild right optic atrophy; the orbital lesions were similar as on previous MRI. She did not improve after IVMP 1 g daily for 5 days, hence, six sessions of plasmapheresis were performed and she improved afterward to walk with frame. Azathioprine and corticosteroid were initiated for prevention of relapses for her NMO-IgG negative NMOSD. As repeated, MRI showed similar orbital masses, right orbital biopsy through anterior orbitotomy was performed 1 year after onset. Histopathological study revealed a circumscribed lymphoid lesion consisting of dense infiltrate of mixed lymphoid cells and plasma cells, the plasma cells were mature-looking and no atypical infiltrate or epithelial component were observed. Immunohistochemistry showed mixtures of B and T cells, the plasma cells showed no light chain restriction. Consistent with the conjunctival biopsy, the overall features were suggestive of reactive lymphoid hyperplasia (Figure 3). She subsequently developed further recurrent myelitis while on azathioprine 75 mg daily and refused increase of azathioprine dose due to concerns about its side effects. MRI brain repeated a year later showed that the nodular lesions were persistent at the superior aspects of bilateral eyeballs with some contrast enhancement, but reduced in size, and repeated serology detected AQP4-IgG by cell-based assay (5). She walked with mild assistance and was stable.

### Patient 2

**Figure 2:**
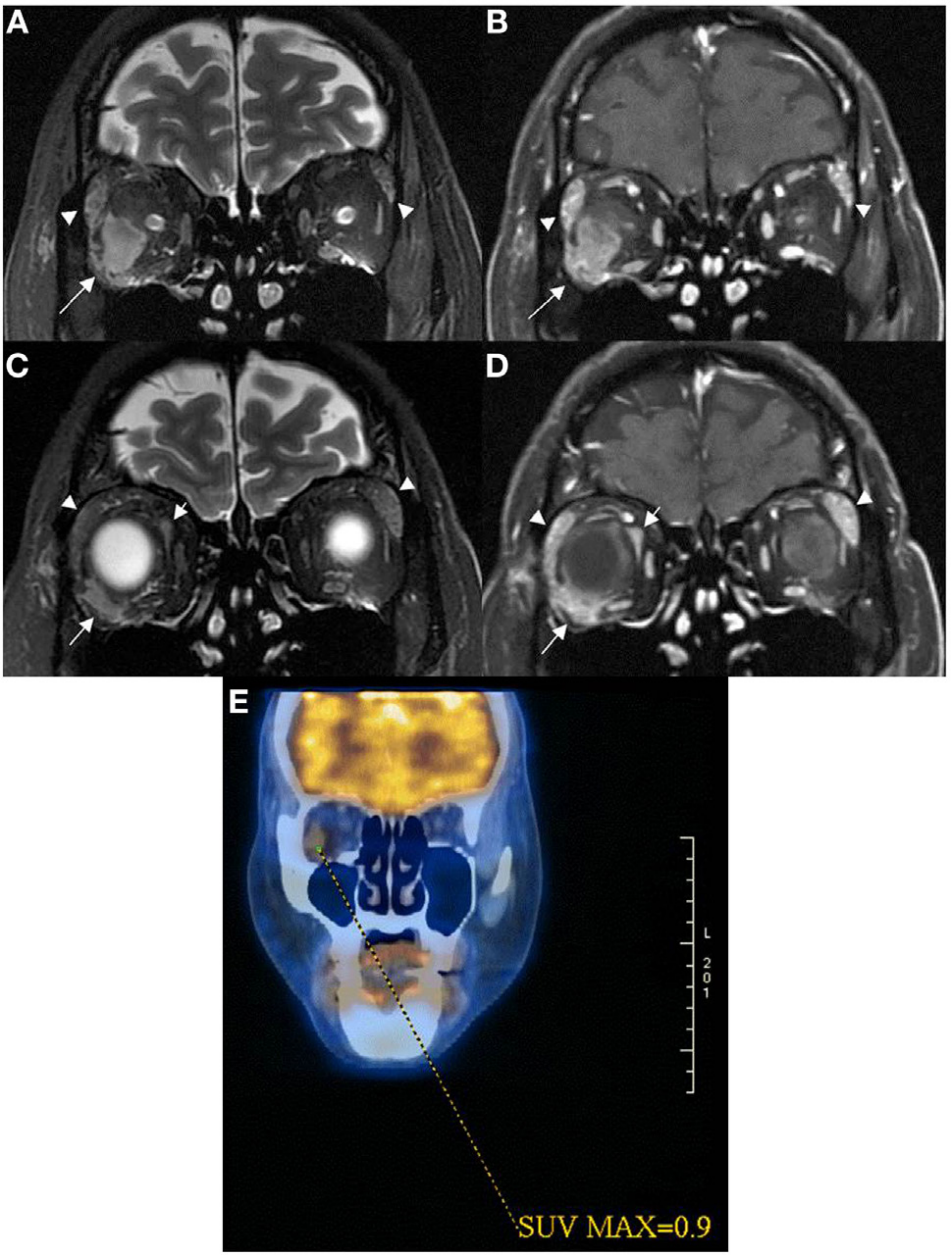
Coronal MRI of patient 2 showed intraconal orbital mass lesion (arrow) with low T2W signal **(A)** and modest contrast enhancement **(B)** having a 0.9 SUV on ^18^FDG PET image **(E)**. Coronal MRI more anteriorly showed extraconal orbital lesions (short and long arrows) with low T2W signals **(C)** with modest contrast enhancement **(D)**. One of the extraconal lesion infiltrated the outer lateral part of the right globe [long arrows in **(C,D)**]. Lacrimal glands were enlarged [arrowheads in **(A–D)**], and they demonstrated low T2W signals with modest contrast enhancement suggestive of lymphoid tissue infiltrate.

A 43-year-old Chinese woman with good past health presented with rapidly progressive bilateral painless visual loss over 2 days followed by bilateral lower limb weakness 4 days later. Examination revealed bilateral blindness with papilledema and paraplegia. Urgent MRI orbits and brain revealed no cerebral parenchymal lesion but right orbital mass (Figure 2), MRI spine performed 1 week later showed diffuse T2W hyperintense lesions with contrast enhancement at C3/4, C6-T1, T2-T4, and T7-T9 levels. LP revealed opening pressure of 14 cm water and CSF analysis yielded protein 0.75 g/L, glucose 4.7 mmol/L, and raised IgG level without OCB or malignant cells. Her serum IgG, IgM, complement C3 and C4 levels were normal, but IgA was elevated to 477 mg/dL (normal 70–386 mg/dL) without monoclonal band. Her serum was positive for AQP4-IgG by cell-based assay and ANA at low titer (1/160) without elevated anti-dsDNA level. Biopsy of the right orbital mass *via* orbitotomy 4 days after symptoms onset revealed that the mass consisted of fibrous tissue with lobules of lymphoid infiltrate separated by fibrous septa; the infiltrate showed many GC surrounded by dense sheets of small lymphocytes (Figure 4). High-endothelial venules (HEV) and scattered large activated lymphoid cells were observed between the GC. Marginal zone proliferation was not discerned and a small number of mature plasma cells were seen patchily. There were no sheets of large lymphoid cells, Hodgkin cells, or variants. Immunohistochemistry confirmed the GC were reactive (BCL2^−^CD10^+^BCL6^+^) and positive for B cell marker CD20 (Figure 4); there was expansion of the mantle zone but with moderate number of T lymphocytes in between the follicles, and the B cells in the mantle zone were negative for CD5 and CD43. The lymphoid cells were negative for cyclinD1 (not supportive of mantle zone lymphoma). The T lymphocytes were CD2^+^3^+^5^+^7^+^ and the CD4:CD8 ratio was around 5:1. There were no increased natural killer cells on CD56 staining. The plasma cells were prototypical for kappa and lambda light chain, and a small number of plasma cells were positive for IgG4 (IgG4/IgG ratio ~15%) (Figure 4). Although the expanded mantle zone was atypical, the B cells in the mantle zone were negative for CD5, CD43, and cyclin D1, hence not supportive of mantle cell lymphoma. Biopsy of the right lacrimal gland revealed benign gland with patchy reactive lymphoid infiltrate, characterized by preserved acinar architecture, benign lobules with patchy mild lymphocytic, and mature plasma cell infiltration.

**Figure 3:**
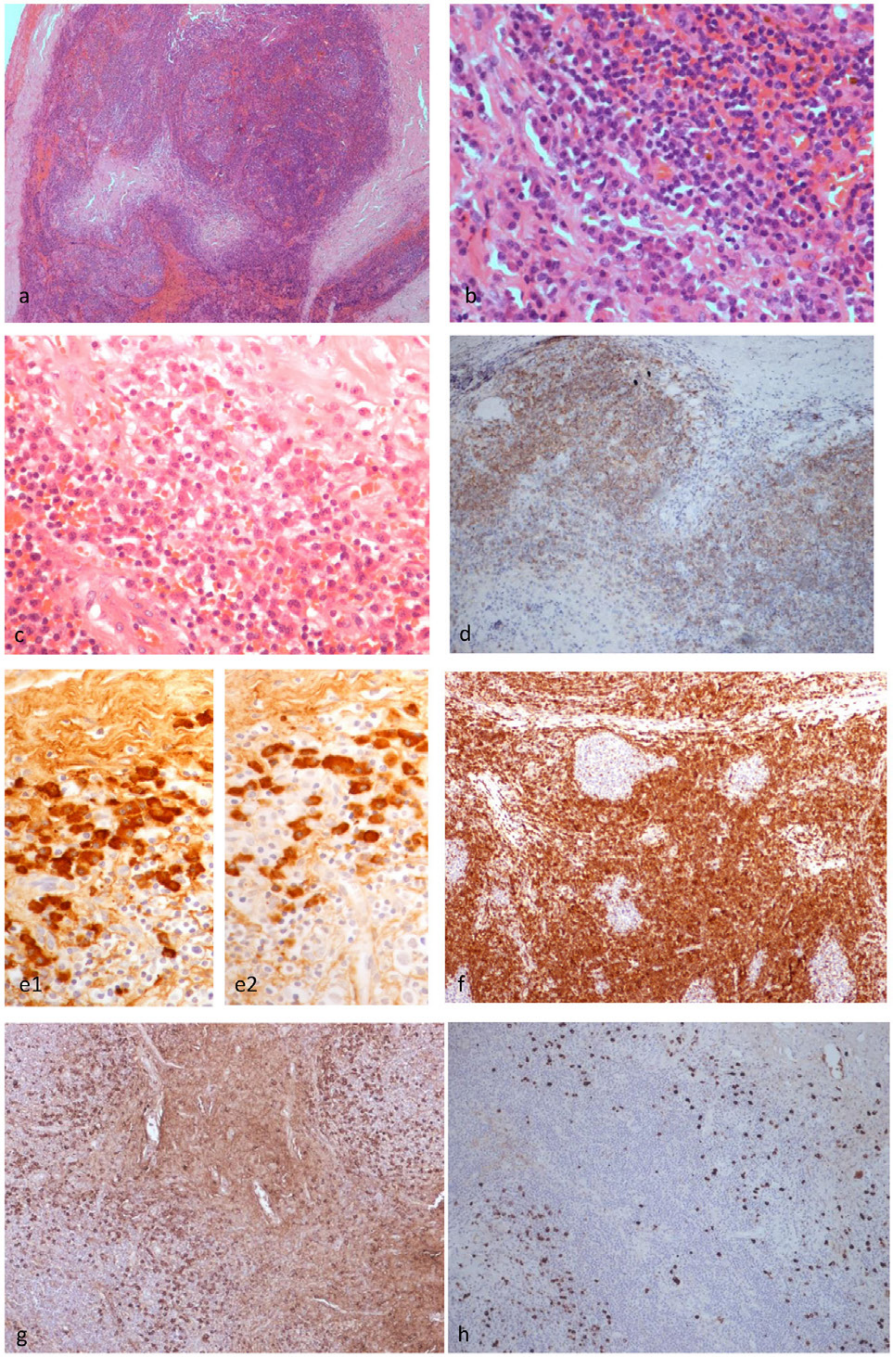
Histopathological findings of patient 1. The left upper eyelid mass showed cellular lymphoid nodules separated by dense hyalinized fibrous septa (H&E ×4) **(A)**. The nodules were composed of hyperplastic lymphoid follicles surrounded by well formed mantle zone and the septa contains mature plasma cells (H&E ×40) **(B)**. Mature plasma cells were shown in another area of the septa (H&E ×40) **(C)**. CD20 monoclonal antibody staining revealed that the B cells were confined within the follicles (×10) **(D)**. The plasma cells are polytypic for kappa **(e1)** and lambda **(e2)** light chain (×10). BCL2 antibody staining revealed the absence of BCL2 in the germinal centers **(F)**. Many of the plasma cells are positive for IgG **(G)** and a fair amount are positive for IgG4 **(H)** (×10), but the IgG4:IgG ratio is less than 40%.

**Figure 4:**
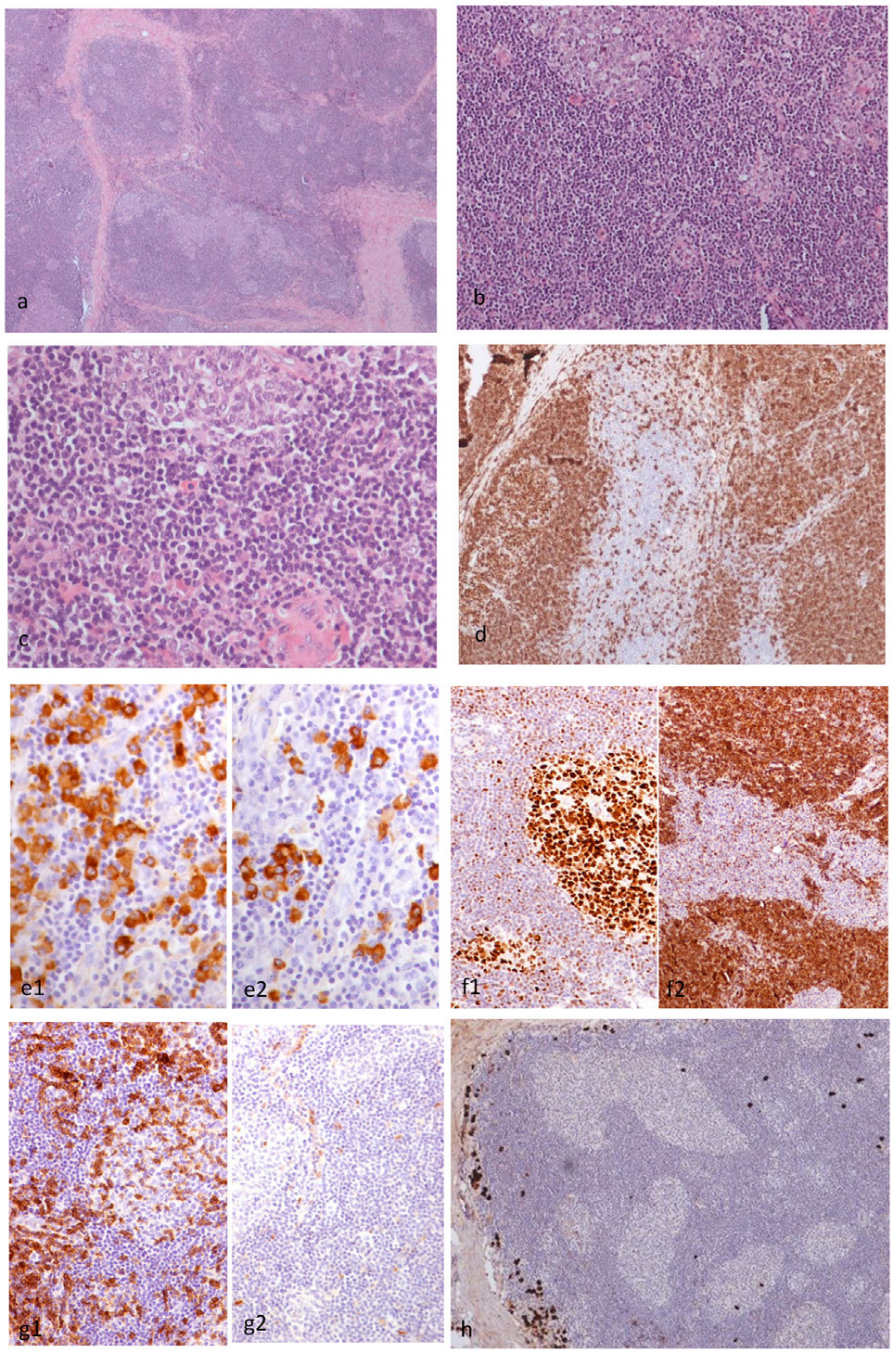
Histopathological findings of patient 2. The right orbital mass consisted of cellular lymphoid nodules separated by hyalinized fibrous septa, but in areas, these nodules were coalescing (H&E ×4) **(A)**. The nodules showed reactive germinal centers (GC) surrounded by small-sized lymphocytes (H&E ×20) **(B)**. The lymphocytes did not show cytologic atypia (H&E ×40) **(C)**. CD20 monoclonal antibody staining revealed that the lymphoid nodules consisted of B cells, and the interfollicular B cells were within normal limits (×10) **(D)**. The plasma cells are polytypic for kappa (e1) and lambda (e2) light chain (×10) **(E)**. Staining with BCL6 and BCL2 antibodies revealed the presence of BCL6 (f1) and absence of BCL2 (f2) in the GC **(F)**. Staining with CD5 and cyclinD1 antibodies revealed that the B cells in the mantle zone were negative for both CD5 (g1) and cyclinD1 (g2) in the GC **(G)**. A small number of IgG4 positive plasma cells surround the hyperplastic lymphoid follicles (×10) **(H)**.

She was treated with IVMP 1 g daily for 5 days followed by plasmapheresis. Azathioprine and prednisolone (1 mg/kg) were then initiated to prevent relapse. She had improvement in vision and lower limb power to grade 3-/5. Azathioprine was withdrawn due to liver function derangement and replaced by mycophenolate mofetil 1 g BD. Three months after initial presentation, she developed recurrent myelitis affecting T6–9 and conus medullaris regions. She was treated with IVMP 1 g daily for 5 days and plasmapheresis. Prednisolone was gradually tailed down to 15 mg daily. She improved gradually to walk with frame 4 months later. Whole-body PET-CT scan performed 4 months after clinical onset revealed two small eumetabolic enhancing soft tissue masses without significant fluorodeoxyglucose uptake in the right orbit (Figure 2E), and two prominent eumetabolic cervical lymph nodes, which could be reactive; there was no hypermetabolic or destructive lesion. She developed further relapse of severe myelitis affecting C1–6 5 months after the last attack. Repeated MRI brain revealed no parenchymal lesions and the intraconal lesions of right orbit were static and persistent with homogenous enhancement. Her vision and left upper limb power improved after IVMP and plasmapheresis, but she remained paraplegic. MMF was increased to 1.5 g BD and she remained stable; however, 4 months later, she decided to switch to rituximab for potentially superior efficacy. Rituximab 375 mg/m^2^ weekly for 4 weeks was intiatied and MMF was tailed down to 1 g BD a month later. Unfortunately, she developed fulminant relapse of cervical myelitis at C1–2 region with respiratory failure 2 months later. Despite aggressive treatment including IVMP 1 g daily for 5 days and plasmapheresis, she succumbed from complicating pneumonia.

Written informed consent was obtained from the participants for the publication of this case report.

## Discussion

B cells play important roles in the pathogenesis of AQP4-IgG-positive NMOSD as their progeny, plasmablasts, and plasma cells, produce the pathogenic autoantibodies (8, 9). In addition, the efficacy of B cell depletion by anti-CD20 monoclonal antibody (rituximab) in a significant proportion of NMOSD patients implies that other B cell functions besides autoantibody production are important in NMOSD pathophysiologies as mature plasmablasts and plasma cells do not express CD20 (8, 9, 12). Antigen presentation to T cells and secretion of proinflammatory cytokines by B cells are likely important pathogenetic mechanisms in NMOSD (8).

Specific humoral immunity requires B cells contact with the specific antigen and interaction with helper T cells, this occur with organization of B and T cells into GC where T and B cells interact to allow development of long-lived memory B cells and plasma cells capable of producing high-affinity specific antibodies (13). In GC, chemokines and cytokines secreted from activated T cells, typically T follicular helper (TFH) cells, drive maturation and differentiation of B cells with somatic hypermutation, class switching, and affinity maturation of immunoglobulins (14, 15). Our two patients had orbital mass lesions on presentation of LETM or ON rapidly followed by LETM, which were shown to be ELF containing GC. ELF are structurally similar to secondary lymphoid organs including encapsulated organs such as the spleen and lymph nodes, and mucosal-associated lymphoid tissue (MALT) such as the tonsils and Peyer’s patches. ELF occur in sites of chronic inflammation typically mucosal sites such as inducible gut-associated lymphoid tissue and bronchus-associated lymphoid tissue. ELF are characterized by development of HEV, aggregates of T and B cells often with T/B segregation and, in the majority, follicular dendritic cell networks (16). Abundant antigen-presenting cells including B cells are found around HEV, suggesting that development of HEV contributes to ectopic GC formation by recruitment of peripheral blood B cells and antigen-presenting cells (such as dendritic cells) to the ELF (13). ELF tend to be transient structures that are triggered by infection or immunization, which often resolve after antigen clearance (16). In autoimmune diseases, ELF most commonly are found in chronic inflammation and function to maintain the autoimmune pathophysiologies (17). Ectopic GC are found in inflamed tissues of autoimmune diseases including thymuses of MG, synovial tissue of rheumatoid arthritis, salivary glands of Sjogren’s syndrome, thyroid of Hashimoto’s thyroiditis, and meninges of secondary progressive multiple sclerosis (16–18).

Our first patient had long history of glaucoma and right eye puffiness for 2 years associated with conjunctival mass before presentation with LETM. Her conjunctival mass showed reactive lymphoid hyperplasia histologically. This raises the possibility of glaucoma-related ocular surface disease with chronic inflammation in the conjunctiva-associated lymphoid tissue (CALT) resulting in the hyperplastic lymphoid follicles. The frequency of orbital masses containing ELF detected in glaucoma or other ophthalmological diseases is unknown. CALT is physiologically a form of MALT described as eye-associated lymphoid tissue (EALT) (19). Various types of insults to the surface of the eye can lead to ocular surface diseases (OCDs) (20). OCDs are characterized by inflammation of the ocular surface with activation of immune cells in CALT contributing to numerous inflammatory cells infiltration, remodeling of the ocular surface epithelia, and EALT components (20). Glaucoma is a chronic progressive optic neuropathy characterized by irreversible loss of retinal ganglion cells and is known to cause OCD, described as glaucoma-related OSD (19). In addition, glaucoma-mediated optic nerve damage might lead to release of AQP4 and hence contribute to the development of AQP4 autoimmunity in our first patient.

Importantly, the first patient had right optic atrophy on MRI and prolonged right VEP suggestive of previous subclinical ON and the second patient had severe ON on presentation. We hypothesize that for both patients, previous subclinical ON led to optic nerve damage with release of AQP4 to adjacent inflamed regions as optic nerve expresses high level of AQP4. This is followed by formation of ELF with ectopic GC around the eyeballs under the effects of proinflammatory cytokines and chemokines released during ON. Important cytokines and chemokines involved in the formation of ELF containing GC include IL-17, IL-21, IL-22, IL-27, CXCL13, CCL19, CCL21, and CXCL12 (16, 21–25), which play key roles in migration of antigen-presenting cells (dendritic cells), B and T cells into the ELF, aggregation of B and T cells and their segregation in different zones of the GC for functioning (21). Follicular dendritic cells play key roles in the formation of ectopic GC as they produce CXCL13 (21), the important chemokine for migration of TFH cells toward B cell follicles as they express high level of CXCR5 and respond to CXCL13 (26). TFH cells belong to a highly specialized CD4^+^ memory T cell subset essential for regulation of B cell activation, antibody affinity maturation, and GC reaction *via* the expression of surface receptors including inducible T-cell costimulator (ICOS) and programme cell death protein 1 (24, 27, 28). IL-21 is the main soluble cytokine released by TFH cells and IL-21/IL-21 receptor signaling in B cells provides strong stimuli for B cell survival, proliferation, and differentiation (29). In addition, Th17 cells have been reported to induce ELF containing GC in the CNS of experimental allergic encephalomyelitis, an animal model of multiple sclerosis (23). This was partly dependent on IL-17, which is also associated with survival and proliferation of B cells (30). CXCL13 and CXCL12 regulate shuttling of B cells within the ectopic GC. CXCL13 predominantly directs B cells to the light zone where antigen selection occurs whereas CXCL12 plays key role in migration of CXCR4^high^ centroblasts to the dark zone where somatic hypermutation of B cell receptor occurs (21, 22). IL-27 exerts inhibitory effect on formation and functioning of ectopic GC in animal models of autoimmune diseases including SLE and RA (30).

This is the first report of ectopic GC observed in AQP4-IgG positive NMOSD patients. Considering the presence of either active or chronic ON in the two patients on initial presentation, the orbital ectopic GC, which were in close proximity to the optic nerves in our two patients might be related to the development of AQP4 autoimmunity with production of AQP4-IgG and their severe myelitis and ON. Current evidence supports that in autoimmune diseases, ELF containing GC mostly develop in the context of chronic inflammation and contribute to maintain the disease process by acting as actively functioning GC where an antigen-driven selection process allows affinity maturation of B cells and differentiation to plasma cells capable of secreting high-affinity autoantibodies specific for the autoantigens (21, 31). B cells of ELF in autoimmune diseases display highly somatically hypermutated immunoglobulin VH and VL regions consistent with a local antigen-driven process, and clonal diversification and differentiation to autoantibody-producing cells have been confirmed to occur within ELF by lineage tree analysis of immunoglobulin gene repertoire of B cells and plasma cells from ELF in autoimmune diseases (32–34). In addition, B cells within ectopic GC express activation-induced cytidine deaminase, the enzyme regulating somatic hypermutation and class switching of the immunoglobulin genes (16). ELF containing GC in autoimmune diseases are often associated with a more severe disease course (21, 31), which was observed in our two patients. It is possible that plasma cells in the ectopic GC of our two patients contained autoreactive plasma cells capable of secreting high-affinity AQP4-IgG. A recent study reported that the percentages and numbers of circulating memory TFH cells (CCR7^−^ICOS^+^, CCR7^+^ICOS^+^) and levels of IL-21 in plasma and CSF were significantly higher in NMOSD patients compared to healthy subjects (35). In addition, the percentages CCR7^−^ICOS^+^ memory TFH cells were positively correlated with annualized relapse rate, plasma IL-21, and AQP4-IgG levels whereas the percentages of CCR^+^ICOS^+^ memory TFH cells were positively correlated with CSF white cell counts, protein, and IL-21 levels. These findings suggest that circulating memory TFH cells may participate in relapse and development of NMOSD and be target of novel therapies (35).

Our findings suggest the possibility that development of AQP4 autoimmunity, the underlying pathogenetic mechanism for AQP4-IgG positive NMOSD in our two patients, involves ectopic GC in their orbital and conjunctival ELF. Our observations may have clinical implication as the potential importance of the ectopic GC in disease pathophysiologies imply that therapies that inhibit the formation and functioning of ectopic GC may be efficacious, such as blockade of IL-21 signaling by monoclonal antibodies or IL-21 receptor antagonists; especially both patients had severe myelitis and/or ON, and the second patient did not respond to rituximab.

## Ethics Statement

Institutional Review Board of the University of Hong Kong/Hospital Authority Hong Kong West Cluster (HKU/HA HKW IRB).

## Author Contributions

KC managed the patient and wrote up the manuscript. RL performed MRI brain and cord scan and reviewed all the imaging findings in details. KL is involved in care of the patients and contributed to manuscript writing. FL examined the biopsied tissues, wrote the pathology report, and prepared the photos.

## Conflict of Interest Statement

The authors declare that the research was conducted in the absence of any commercial or financial relationships that could be construed as a potential conflict of interest.

## Supplementary Material

Figure S1 Timelines of acute attacks and treatments for patients 1 **(A)** and 2 **(B)**. LETM, longitudinal extensive transverse myelitis; R, right; ON, optic neuritis; IVMP, intravenous methylprednisolone; PLEX, plasma exchange; aza, azathioprine; pred, prednisolone; MMF, mycophenolate mofetil.
