# Supplementary figures and images for: Orbital Ectopic Lymphoid Follicles with Germinal Centers in Aquaporin-4-IgG-Positive Neuromyelitis Optica Spectrum Disorders
Source: Front Immunol. 2018 Jan 16;8:1947. doi: 10.3389/fimmu.2017.01947 (PMC5776022; doi:10.3389/fimmu.2017.01947)

**A**

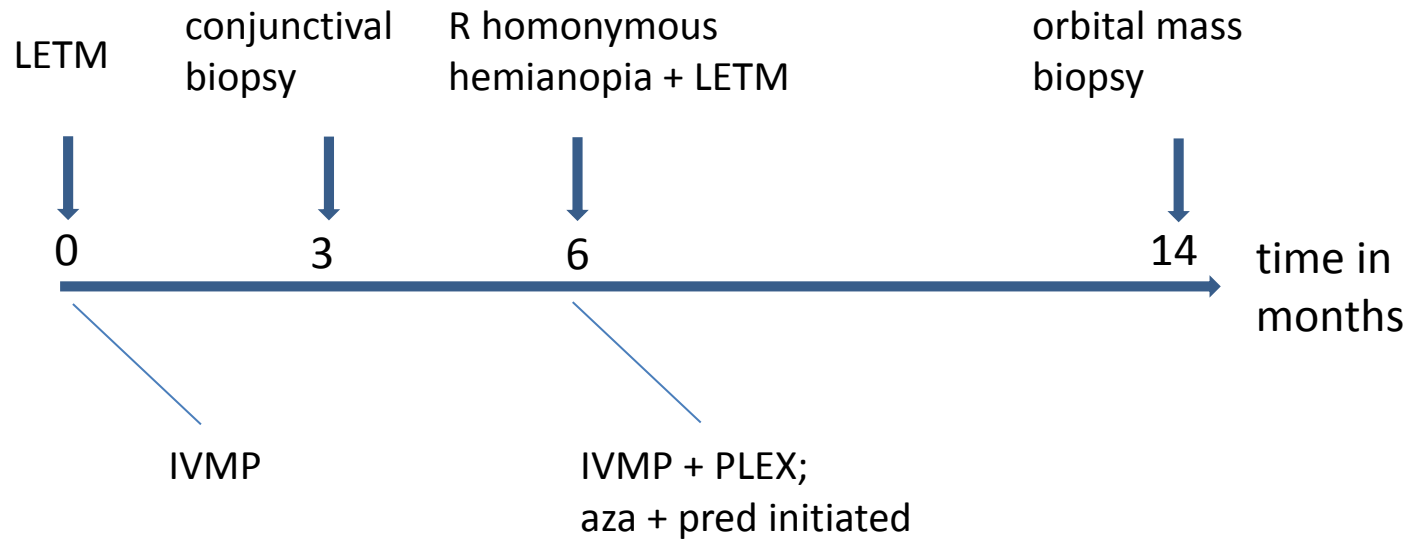

**B**

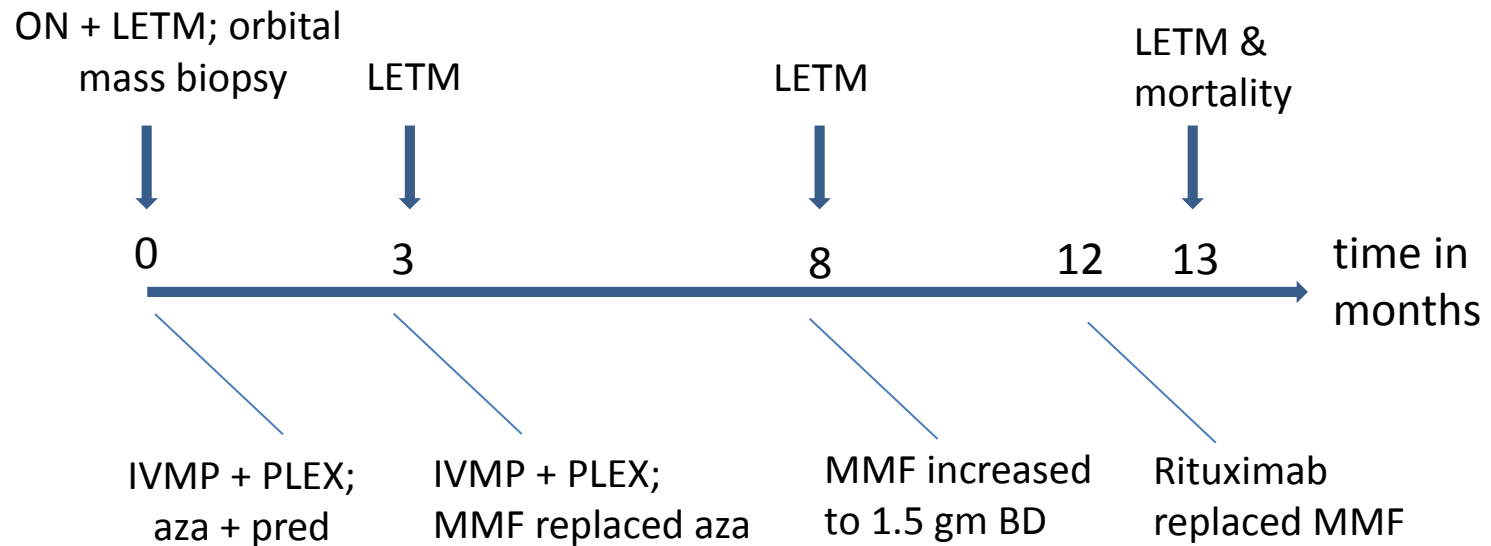

Supplement: Figure S1 — Timelines of acute attacks and treatments for patients 1 (A) and 2 (B). LETM, longitudinal extensive transverse myelitis; R, right; ON, optic neuritis; IVMP, intravenous methylprednisolone; PLEX, plasma exchange; aza, azathioprine; pred, prednisolone; MMF, mycophenolate mofetil. [file image_1.PDF]
